# Overview of systematic reviews - a new type of study. Part I: why and for whom?

**DOI:** 10.1590/S1516-31802012000600007

**Published:** 2013-01-18

**Authors:** Valter Silva, Antonio José Grande, Ana Luiza Cabrera Martimbianco, Rachel Riera, Alan Pedrosa Viegas Carvalho

**Affiliations:** I BSc. Specialist in Cardiac Rehabilitation, Obesity and Statistics and Master’s Student in the Post-graduate Program on Internal Medicine and Therapeutics, Federal University of São Paulo (Escola Paulista de Medicina, Universidade Federal de São Paulo, EPM-Unifesp); Volunteer Research Assistant at the Brazilian Cochrane Centre, São Paulo; Professor at Itapeva Social and Agrarian Sciences College (Faculdade de Ciências Sociais e Agrárias de Itapeva, FAIT), Itapeva, São Paulo, Brazil.; II BSc, MSc. Master’s Student in the Postgraduate Program on Internal Medicine and Therapeutics, Federal University of São Paulo (Escola Paulista de Medicina, Universidade Federal de São Paulo, EPM-Unifesp); Volunteer Research Assistant at the Brazilian Cochrane Centre, São Paulo, Brazil.; III BSc. Specialist in Orthopedics and Master’s Student in the Postgraduate Program on Internal Medicine and Therapeutics, Federal University of São Paulo (Escola Paulista de Medicina, Universidade Federal de São Paulo, EPM-Unifesp); Volunteer Research Assistant at the Brazilian Cochrane Centre and Preceptor at EPM-Unifesp, São Paulo, Brazil.; IV MD, MSc, PhD. Rheumatologist and Professor at Federal University of São Paulo (Escola Paulista de Medicina, Universidade Federal de São Paulo, EPM-Unifesp); Coordinator at Brazilian Cochrane Centre, São Paulo, Brazil.; V BSc, MSc. Specialist in Rehabilitation and Cardiac Physiotherapy and Doctoral Student in the Postgraduate Program on Internal Medicine and Therapeutics, Federal University of São Paulo (Escola Paulista de Medicina, Universidade Federal de São Paulo, EPM-Unifesp); Volunteer Research Assistant at the Brazilian Cochrane Centre, São Paulo, Brazil.

**Keywords:** Review [publication type], Study characteristics [publication type], Decision making, Evidence-based practice, Evidence-based medicine, Revisão, Características dos estudos, Tomada de decisões, Prática clínica baseada em evidências, Medicina baseada em evidências

## Abstract

**CONTEXT AND OBJECTIVE:**

Healthcare decision-making is complex and should involve healthcare professionals, patients and the best level of evidence. The speed of information production creates barriers against keeping up to date. In this light, methodologists have proposed a new type of study: overviews of systematic reviews (OoRs). The aim here was to introduce and demonstrate the role of OoRs in information synthesis for healthcare professionals, managers, researchers and patients.

**DESIGN AND SETTING:**

Time-series study conducted at the Brazilian Cochrane Center, jointly with the Postgraduate Program on Internal Medicine and Therapeutics, Discipline of Emergency Medicine and Evidence-Based Medicine, Department of Medicine, Federal University of São Paulo.

**METHODS:**

To show the growth in the numbers of published papers that provide high-level evidence and thus demonstrate the importance of OoRs for synthesis and integration of information, three filters for study designs were applied to two databases. An equation for predicting the expected number of published papers was developed and applied.

**RESULTS:**

Over the present decade, the number of randomized controlled trials in Medline might reach 2,863,203 and the number of systematic reviews might reach 174,262. Nine OoRs and 15 OoRs protocols have been published in the Cochrane Library.

**CONCLUSIONS:**

With the exponential growth of published papers, as shown in this study, a new type of study directed especially towards healthcare decision-makers was proposed, named “overview of systematic reviews”. This could reduce the uncertainties in decision-making and generate a new hierarchy in the pyramid of evidence.

## RESUMO

**CONTEXTO E OBJETIVO:**

A tomada de decisão em saúde é complexa e deve envolver o profissional de saúde, o paciente e a evidência de melhor nível. A velocidade de geração da informação cria barreiras para manter-se atualizado. Diante disso, metodologistas propuseram um novo tipo de estudo, as *overviews* de revisões sistemáticas (OoRs). O objetivo é introduzir e demonstrar o papel das OoRs na síntese de informações para profissionais da área da saúde, gestores, pesquisadores e pacientes.

**TIPO DE ESTUDO E LOCAL:**

Estudo de série temporal realizado no Centro Cochrane do Brasil, em conjunto com o Programa de Pós-Graduação em Medicina Interna e Terapêutica da Disciplina de Medicina de Urgência e Medicina Baseada em Evidências do Departamento de Medicina da Universidade Federal de São Paulo.

**MÉTODOS:**

Para mostrar o crescimento das publicações que fornecem evidência com alto nível e assim justificar a importância das OoRs na síntese e integração das informações, três filtros para desenhos de estudos foram aplicados em duas bases de dados. Uma equação de predição do número esperado de publicações foi desenvolvida e aplicada.

**RESULTADOS:**

Na presente década, o número de ensaios clínicos randomizados no Medline poderá chegar a 2.863.203 e o número de revisões sistemáticas poderá chegar a 174.262. Nove OoRs e 15 protocolos de OoRs foram publicados na Biblioteca Cochrane.

**CONCLUSÕES:**

Com o crescimento exponencial das publicações, demonstrado neste estudo, um novo tipo de estudo, direcionado especialmente aos decisores em saúde, foi proposto, a OoRs, o qual poderá reduzir incertezas para a tomada de decisão e gerar uma nova hierarquia na pirâmide de evidências.

## INTRODUCTION

The effectiveness of healthcare clinical practice depends on evidence based on higher quality, and practices to be implemented should be discussed between healthcare professionals and their patients, for decision-making.^1^^,^^2^^,^^3^ In order to make this process work, healthcare professionals need to keep up to date. However, this is a complex challenge, given the globalized world and the speed at which information is disseminated.

One example of the complexity of keeping up to date was given by Davidoff et al.^4^ In 1992, it was estimated that healthcare professionals should read 17 to 20 original papers every day to keep up to date in their field. In Medline,^5^ the world’s largest medical library, more than 736,000 new records were published in 2011, with over 21 million citations in PubMed.

An alternative way to reduce the complexity of keeping up to date and facilitate clinical decision-making is to use systematic reviews (SRs). One of the primary functions of this type of study is to summarize clinical information from several studies in order to answer a question relating to diagnosis, prevention or treatment in areas in which the results may or may not be in agreement, through critical evaluation of the evidence.^6^^,^^7^

Taking into consideration only the most important database of SRs, i.e. the Cochrane Library, more than 7,000 articles have been published.^8^ Given this large amount of information, professional updating remains a challenge, even when resorting to SRs. One possible solution to the problem has been put forward by methodologists who are experts on SRs: a new type of study called an overview of systematic reviews (OoRs), considered to be a “friendly front end” for healthcare decision-making.^9^^,^^10^^,^^11^ By definition, an OoRs is a study designed to integrate and produce a synthesis of information from existing SRs on a particular clinical condition, considering all the available interventions for treating or preventing this condition.^9^^,^^10^^,^^11^

For didactic purposes, the present analysis will be published as a series of three articles. Part I, presented here, focuses on the growth of published papers presenting the best level of evidence for decision-making relating to healthcare, thereby justifying the creation of OoRs. Part II, to be presented in a forthcoming article, will describe the state of the art of OoRs from the Cochrane Collaboration. In Part III, a new hierarchical evidence pyramid will be proposed, taking this new type of study into consideration.

## OBJECTIVE

The objective of this study was to introduce and demonstrate the role of OoRs in relation to synthesis of information for healthcare professionals, managers, researchers and patients.

## METHODS

**Chart 1.**
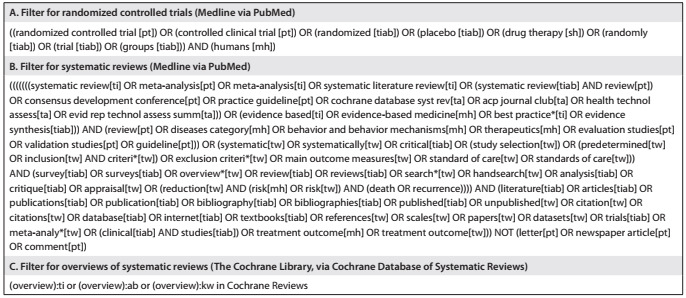
Search filter from study designs used

This was a time-series study that included application of filters to study designs (to select studies that provide the highest level of evidence) in virtual databases, in order to show the growing number of published papers within the field of healthcare that provide high levels of evidence for decision-making and thus seek to demonstrate the importance of OoRs as a type of study that provides a synthesis of the evidence and integrates it. For this, two databases were selected (the Cochrane Library via the Cochrane Database of Systematic Reviews, and Medline via PubMed) and three search filters for study designs were applied. The first filter used (filter A) was the sensitivity-maximizing version of the Cochrane Highly Sensitive Search Strategy^12^ for identifying randomized controlled trials (RCTs) in Medline. The second filter (filter B) was designed to search for all systematic reviews (SRs) in Medline.^13^^,^^14^ The third search filter (filter C) was developed to identify overviews of systematic reviews (OoRs) in the Cochrane Library. The three search filters used are shown in Chart 1.

**Figure 1.**
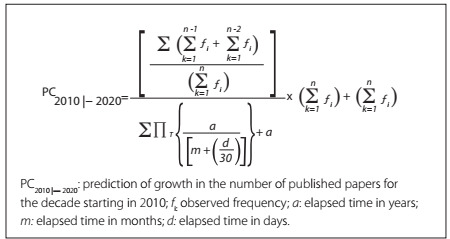
Equation for predicting the expected number of published papers.

Subsequently, an equation to predict expected future numbers of published papers was modeled (designed). This equation was based on the fraction of the present decade that has already elapsed, in order to determine the cumulative frequency for the period. The equation thus developed is shown in Figure 1**.**

## RESULTS

**Figure 2.**
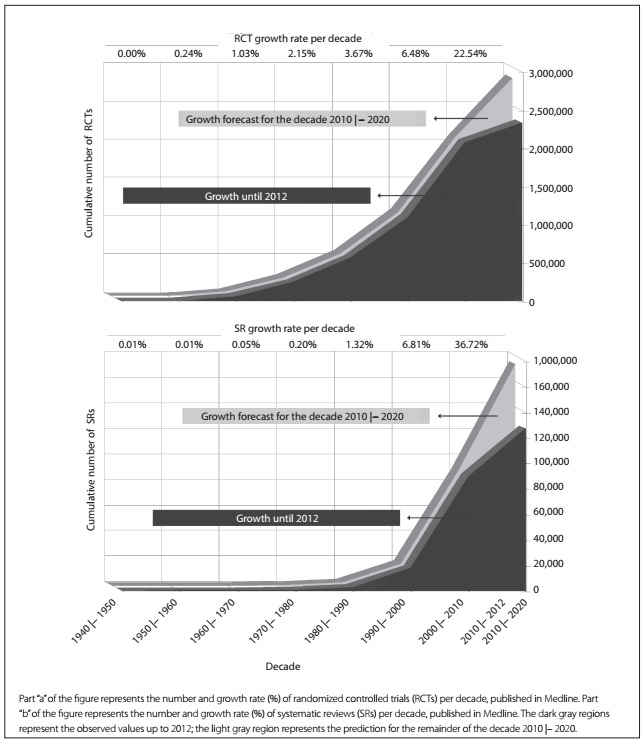
Growth in the numbers of published papers in Medline with a high level of evidence for healthcare decision-making.

Up to the cutoff date of May 21, 2012, more than two million RCTs (n = 2,336,617) were identified by applying filter A (Figure 2, part “a”, in dark gray). The forecast growth was taken to be based on the number of studies published over the date range of the search. Thus, over the decade between 2010 and 2020, the number of RCTs in Medline might reach 2,863,203 (Figure 2, part “a”, in light gray). 263,002 RCTs were published in the decade beginning in 2010, up to the cutoff date considered, but more than 526,586 RCTs are expected over the remainder of the decade, thus resulting in almost 800,000 new RCTs published in Medline over the entire decade 2010-2020.

Up to the cutoff date of June 6, 2012, more than 100,000 SRs (n = 127,044) were identified by applying filter B to the Medline database (Figure 2, part “b”, in dark gray). The forecast growth was taken to be based on the number of studies published over the date range of the search. Thus, over the decade between 2010 and 2020, the number of SRs in Medline might reach 174,262 (Figure 2, part “b”, in light gray). 37,785 SRs were published in the decade beginning in 2010, up to the cutoff date considered, but more than 47,281 SRs are expected over the remainder of the decade, thus resulting in more than 85,000 new SRs published in Medline over the entire decade 2010-2020.

By applying filter C, to identify OoRs in the Cochrane Library, 57 studies were identified, but 33 were excluded because they were not OoRs (using a very sensitive but low-specificity search). Thus, up to the cutoff date of June 15, 2012, nine OoRs had been published, and 15 OoRs protocols were in this database **(**Table 1**).**^15^^,^^16^^,^^17^^,^^18^^,^^19^^,^^20^^,^^21^^,^^22^^,^^23^^,^^24^^,^^25^^,^^26^^,^^27^^,^^28^^,^^29^^,^^30^^,^^31^^,^^32^^,^^33^^,^^34^^,^^35^^,^^36^^,^^37^^,^^38^

**Table 1.** Growth of overviews of systematic reviews (OoRs) in the Cochrane Library

Year considered	OoRs protocol % (n)^references^	OoRs % (n)^references^	Both % (n)
2009	4.2 (1)^15^	4.2 (1)^30^	8.3 (2)
2010	12.5 (3)^16-18^	4.2 (1)^31^	16.7 (4)
2011	33.3 (8)^19-26^	20.8 (5)^32-36^	54.2 (13)
2012	12.5 (3)^27-29^	8.3 (2)^37,38^	20.8 (5)
2009 to 2012	62.5 (15)^15-29^	37.5 (9)^30-38^	100 (24)

## DISCUSSION

The information from Medline, which is the world’s largest medical library,^5^ with citations in its database going back to the 1940s, shows that PubMed (http://www.ncbi.nlm.nih.gov/pubmed/) includes over 21 million citations for biomedical literature, comprising journals and books on the life sciences that are online through Medline. In 2011 alone, over 736,000 new references were published and, therefore, to acknowledge all of these new papers would have meant reading about 84 articles per day.

In this study, we chose to analyze the growth in the numbers of published RCTs, SRs and OoRs, because these study designs present the highest levels in the hierarchy of evidence for clinical decision-making.^39^

Medline was chosen as the database to assess because it is considered to be the most important search tool within the field of healthcare.^5^ The Cochrane Library was chosen because it is the largest and most important database of SRs and OoRs with over 7,000 citations.^7^

An evidence-level hierarchy was previously proposed in order to provide the best evidence for decision-making in healthcare.^39^ However, this was a heuristic solution, i.e. it assumed a solution that was close to the ideal, even though such a solution would not necessarily be the best possible solution. Nonetheless, it may be considered satisfactory. In order to achieve a satisfactory hierarchy for evidence levels, the quality of evidence is crucial and should be evaluated.

The quality of published papers may be compromised for a variety of reasons, which may include methodological faults, conflicts of interest, data manipulation and so on. Thus, healthcare professionals not only need to keep up to date but also should know how to judge the quality of evidence. To do this, certain tools are recommended, such as the Cochrane Collaboration’s tool^40^ for assessing RCT risk of bias and the AMSTAR tool for assessing the methodological quality of SRs.^41^

To reduce the uncertainties in healthcare decision-making, all the relevant studies need to be found. Therefore, the search strategy should be highly sensitized, i.e. it should be able to detect Mesh terms (Medical Subject Headings) and free-text terms within titles and abstracts indexed in the databases considered.^11^ This implies that not all RCTs and SRs counted in Figure 2 are actually the types of studies for which the search strategy was developed. This is a possible limitation of the present analysis.

Another point to be noted is in relation to the predicted growth among the types of studies considered in the present analysis. These numbers represent numerical approximations based on the publication behavior of previous decades.

Ecological evidence can be highlighted to explain the ascendency of RCT and SR publication: 1) the vertex of the growth curve of the cumulative number of RCTs occurs between 1970 and 1980 (Figure 2, part “a”), which coincides with historical events (for example, Archibald Cochrane’s warning about professionals’ collective ignorance about healthcare and the first register of RCTs, among others); 2) the vertex of the growth curve of the cumulative number of SRs occurs close to the 1990s (Figure 2, part “b”), which coincides with the birth of the Cochrane Collaboration,^42^ which holds a permanent seat within the World Health Organization, as well as being one of the most important producers of this type of study. Although there is a risk of ecological fallacy in this interpretation, the hypotheses presented show validity of logic.

Despite the limitations noted, the growth in RCTs and SRs has, as seen from the results, been exponential. Because healthcare decision-making is naturally complex, SR methodologists have proposed the use of overviews of systematic reviews,^8^^,^^9^^,^^10^ which have also shown growth in the number of published papers.

In Part II of this series of three papers, the state of art of overviews of systematic reviews and details of study design will be presented.

## CONCLUSIONS

Keeping up to date remains a challenge, considering the great quantity and varying quality of information available. Thus, SR methodologists have proposed a new type of study especially suited for healthcare decision-makers, named “overview of systematic reviews”. This new type of study was developed to provide a synthesis and integrate information from multiple studies in order to reduce the uncertainties in decision-making. This may generate a new hierarchy in the pyramid of evidence.
